# mRNA and miRNA expression profiles in an ectoderm-biased substate of human pluripotent stem cells

**DOI:** 10.1038/s41598-019-48447-z

**Published:** 2019-08-15

**Authors:** Shuuji Mawaribuchi, Yasuhiko Aiki, Nozomi Ikeda, Yuzuru Ito

**Affiliations:** Biotechnology Research Institute for Drug Discovery, National Institute of Advanced Industrial Science and Technology (AIST), Central 5, 1-1-1 Higashi, Tsukuba, Ibaraki 305-8565 Japan

**Keywords:** Stem-cell differentiation, Embryonic stem cells

## Abstract

The potential applications of human pluripotent stem cells, embryonic stem (ES) cells, and induced pluripotent stem (iPS) cells in cell therapy and regenerative medicine have been widely studied. The precise definition of pluripotent stem cell status during culture using biomarkers is essential for basic research and regenerative medicine. Culture conditions, including extracellular matrices, influence the balance between self-renewal and differentiation. Accordingly, to explore biomarkers for defining and monitoring the pluripotent substates during culture, we established different substates in H9 human ES cells by changing the extracellular matrix from vitronectin to Matrigel. The substate was characterised by low and high expression of the pluripotency marker R-10G epitope and the mesenchymal marker vimentin, respectively. Immunohistochemistry, induction of the three germ layers, and exhaustive expression analysis showed that the substate was ectoderm-biased, tended to differentiate into nerves, but retained the potential to differentiate into the three germ layers. Further integrated analyses of mRNA and miRNA microarrays and qPCR analysis showed that nine genes (*COL9A2*, *DGKI*, *GBX2*, *KIF26B*, *MARCH1*, *PLXNA4*, *SLC24A4*, *TLR4*, and *ZHX3*) were upregulated in the ectoderm-biased cells as ectoderm-biased biomarker candidates in pluripotent stem cells. Our findings provide important insights into ectoderm-biased substates of human pluripotent stem cells in the fields of basic research and regenerative medicine.

## Introduction

Cell therapies can target various human diseases and regenerative medicine applications through the use of different cell lines, including neural stem cells, mesenchymal stem cells, hematopoietic stem cells, and differentiated cells from embryonic stem (ES) cells and induced pluripotent stem (iPS) cells^1–3^. In particular, both ES cells and iPS cells have broad applications in various disease states^2,3^. However, the pluripotency levels and differentiation propensities of these cells vary dramatically^4–6^. In addition, cell culture conditions, such as growth media, extracellular matrices, and environmental cues, influence the balance between self-renewal and differentiation^7–9^. Recently, Allison *et al*. reported that *GATA6*-positive ES cells exhibit an endoderm-biased pluripotent substate^10^. Therefore, precisely defining and monitoring the pluripotent substates of ES and iPS cells is important for basic research and clinical applications.

ES and iPS cells are powerful resources for regenerative medicine; thus, biomarkers play important roles in defining and monitoring cell evaluation during culture^2,3^. To identify biomarkers and molecular targets for disease diagnosis and cell state evaluation by investigating exhaustive expression pattern, DNA microarray, RNA-seq, microRNA (miRNA) microarray, and proteomics are novel and robust technologies^7,11–14^. Exosomes are major extracellular vesicles produced in the endosomal compartment of cells^15^. miRNAs within cells could be released extracellularly by exosomes or cell damage^16–18^. The miRNAs found in exosomes and damaged cells derived from the serum, plasma, urine, and culture medium are expected to be useful as noninvasive biomarkers^16–18^. Furthermore, the integration of miRNA target prediction and mRNA transcriptomics approaches may provide new candidates for high-quality and reliable biomarkers^19,20^.

Few reports have described biomarkers that can be used to evaluate pluripotency during the earliest stages of differentiation and in germ layer-biased substates in human pluripotent stem cells^10^. Accordingly, to explore biomarkers for defining and monitoring the pluripotent substates during culture, we established different substates in H9 human ES cells by changing the extracellular matrix from vitronectin to Matrigel. We then evaluated the expression patterns of various markers, examined the differentiation potential of the cells, and performed integrated analyses of mRNA and miRNA microarrays of the cells and exosomes to explore high-quality and reliable biomarkers. Our findings provided insights into the use of biomarker candidates during the earliest stages of ectodermal differentiation.

## Results and Discussion

### Changes in the extracellular matrix induced two different substates in H9 cells

**Figure 1:**
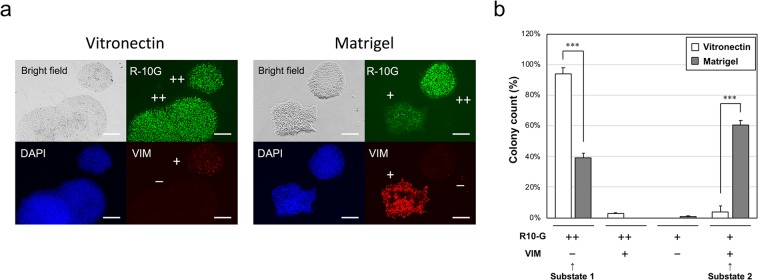
Expression pattern analysis of R-10G epitope and vimentin in H9 cells grown on vitronectin and Matrigel. (**a**) Immunohistochemistry with no permeabilisation using antibodies for a pluripotency marker (R-10G epitope) and a mesenchymal marker (vimentin [VIM]). Nuclei were counterstained with DAPI. The expression levels of R-10G epitope and VIM were classified as “++” and “+” or “+” and “−”. The scale bar represents 200 µm. (**b**) Colony distributions for R-10G epitope and VIM expression patterns. The colonies were manually counted (n = 100, in triplicate). Values are the means ± standard errors. *** indicates *P* < 0.001 by unpaired two-tailed t-tests. R-10G++/VIM− and R-10G+/VIM + colonies were classified as “substate 1” and “substate 2”, respectively.

Pluripotent stem cells require a balance between self-renewal and differentiation, and various factors (e.g., growth media, extracellular matrices, and environmental cues) are crucial for the maintenance of pluripotency^8,9^. To establish techniques for quality evaluation of human pluripotent stem cells using biomarkers, we first examined the conditions for pluripotency in H9 human ES cells by changing the extracellular matrix. H9 cells cultured in TeSR-E8 on Vitronectin XF were transferred to Matrigel. Typical colonies were observed under both conditions, and colonies with wide intercellular spaces appeared under Matrigel conditions, as observed by bright-field microscopy (Fig. 1a). The epithelial-to-mesenchymal transition is observed during ES/iPS cell differentiation^21^. Accordingly, we performed immunocytochemistry with no permeabilisation using antibodies for the pluripotency marker R-10G epitope and the mesenchymal marker vimentin (Fig. 1a)^21,22^. Under vitronectin conditions, the R-10G epitope was highly expressed on the cell surfaces of almost all colonies. A few colonies showed vimentin expression. Under Matrigel conditions, colonies similar to those under vitronectin conditions showed strong expression of the R-10G epitope and almost no expression of vimentin. In contrast, colonies with wide intercellular spaces showed weak expression of R-10G epitope and strong expression of vimentin.

We then counted the colony numbers according to staining categories (Fig. 1b). The percentages of R-10G ++/vimentin− colonies under vitronectin and Matrigel conditions were 94.0% and 38.8%, respectively. R-10G + and vimentin + colonies, which may represent decreased pluripotency compared with R-10G ++ and vimentin− colonies, clearly increased to 60.4% under Matrigel conditions, showing a greater increase than that under vitronectin conditions. Thus, changes in the extracellular matrix from vitronectin to Matrigel induced two different substates. These results also indicated that there was an inverse correlation between R-10G epitope and vimentin expression. Vitronectin XF consists of recombinant human vitronectin protein, whereas Matrigel consists of extracellular matrix proteins, including laminin, collagen IV, heparin sulphate proteoglycans, entactin/nidogen, and growth factors. By changing the extracellular matrix, various factors in Matrigel may affect H9 cells. Importantly, Li *et al*. suggested that Matrigel induces ectoderm differentiation of embryoid bodies^23^. Matrigel is known to be suitable for pluripotent stem cell culture under feeder-free conditions^24^, whereas changing the extracellular matrix should be performed more carefully. Based on bright-field and immunohistochemical observations, we designated R-10G ++ and vimentin− typical colonies as “substate 1” and R-10G + and vimentin + colonies with wide intercellular spaces as “substate 2”.

### Substate 1 and substate 2 cells showed reversibility

**Figure 2:**
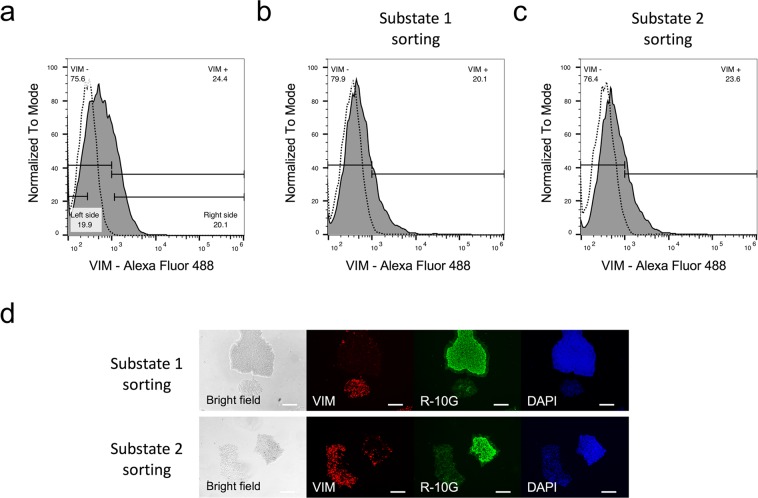
Flow cytometry and immunohistochemical analyses in the H9 cell cultured on Matrigel. (**a**–**c**) Histograms of the H9 cells incubated with anti-vimentin antibodies (solid line) or isotype control (dash line). The substate 1 and substate 2 cells were separated from approximately 20% on the left and right side of vimentin signals, respectively (**a**). Flow cytometry analyses of substate 1 and substate 2 passaged one time after sorting (**b**,**c**). Data are representative of three independent experiments. (**d**) Immunohistochemistry with no permeabilisation using antibodies for a pluripotency marker (R-10G epitope) and a mesenchymal marker (vimentin [VIM]). Nuclei were counterstained with DAPI. The scale bar represents 200 µm.

To investigate the reversibility between substate 1 and substate 2 cells, we examined whether substate 1 and substate 2 cells produced substate 2 and substate 1 cells, respectively. We performed live cell sorting of substate 1 and substate 2 cells by flow cytometry analysis using anti-vimentin antibodies. Substate 1 and substate 2 cells were separated from about 20% on the left and right sides of vimentin signals, respectively (Fig. 2a). Substate 1 and substate 2 cells were subcultured after sorting and were then used for flow cytometry analysis. The subcultured substate 1 and substate 2 cells consisted of both vimentin + and vimentin− cells (Fig. 2b,c). The subcultured cells were also immunostained using anti-R-10G and anti-vimentin antibodies. Both substate 1 and substate 2 cells produced R-10G ++/vimentin− and R-10G +/vimentin + colonies (Fig. 2d). These results indicated that substate 2 cells did not deviate irreversibly from substate 1 cells and that substate 1 and substate 2 cells could show reversibility of their substates.

### Substate 2 cells showed decreased expression of some cell surface markers related to pluripotency

**Figure 3:**
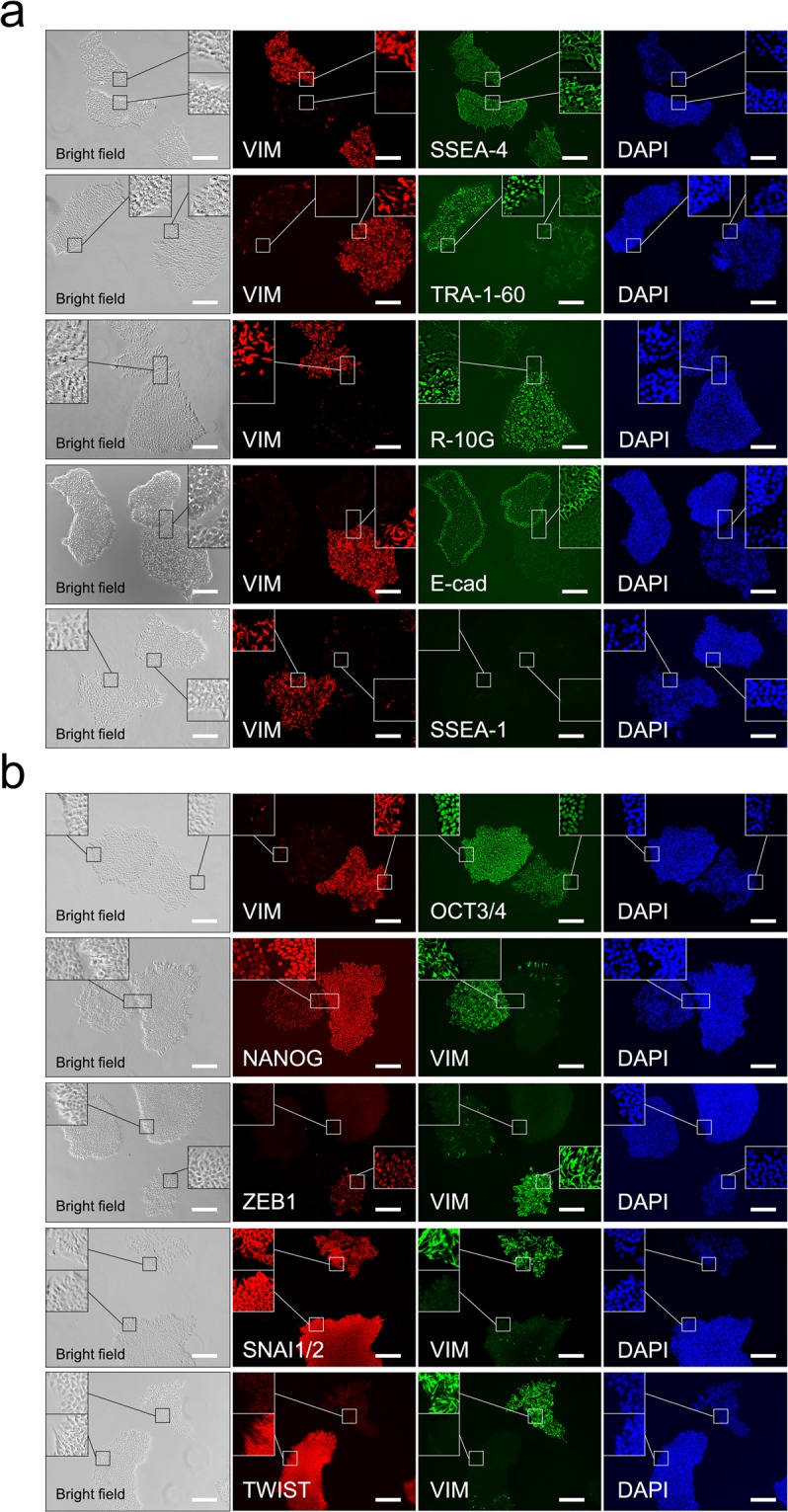
Immunohistochemical analysis of pluripotency, differentiation, and epithelial-to-mesenchymal transition markers in H9 cells cultured on Matrigel. (**a**) Immunohistochemistry with no permeabilisation using antibodies for vimentin (VIM), SSEA-4, TRA-1-60, R-10G, E-cadherin (E-cad), and SSEA-1. (**b**) Immunohistochemistry with permeabilisation using antibodies for VIM, OCT3/4, NANOG, ZEB1, SNAI1/2, and TWIST. Nuclei were counterstained with DAPI. The inset shows a 3 × enlargement of the figure. The scale bar represents 200 µm.

To characterise the two substates of H9 cells maintained on Matrigel with TeSR-E8 medium, we next performed immunocytochemistry with no permeabilisation using antibodies for cell surface markers of pluripotency and differentiation (Fig. 3a). The vimentin− and vimentin + colonies indicated substate 1 and substate 2 cells, as shown in Fig. 1, respectively (Fig. 3a). The cell surface markers SSEA-4 and TRA-1-60 characterise pluripotency, and the marker SSEA-1 characterises early differentiation in ES cells^25^. The epithelial marker E-cadherin has important functions in maintaining the pluripotency of iPS and ES cells^26^. Notably, the SSEA-4 signal was observed in both substate 1 and substate 2 colonies and could not be used to distinguish the substates (Fig. 3a). The TRA-1-60 signal was strong in substate 1 colonies and weak in substate 2 colonies, similar to the R-10G signal (Fig. 3a). E-cadherin was expressed only in substate 1 colonies but not in substate 2 colonies (Fig. 3a). The SSEA-1 signal for early differentiation was not detected in both substate 1 and substate 2 colonies (Fig. 3a), indicating that substate 2 cells were barely differentiated, as shown in Fig. 2. However, the expression patterns of the cell surface pluripotent markers SSEA-4, TRA-1-60, R-10G, and E-cadherin were different in substate 1 and substate 2 colonies.

### ZEB1 was specifically expressed in the nucleus of substate 2 cells

Next, we performed immunocytochemistry with permeabilisation using antibodies for transcription factors related to pluripotency and the epithelial-to-mesenchymal transition (Fig. 3b). Oct3/4 and Nanog are critical transcriptional regulators underlying pluripotency in ES cells^27,28^. OCT3/4 and NANOG were expressed in the nuclei of both substate 1 and substate 2 cells (Fig. 3b). The expression levels were slightly weaker in substate 2 cells than in substate 1 cells. The transcription factors ZEB, SNAI, and TWIST are known to contribute to the epithelial-to-mesenchymal transition^21^. SNAI1/2 was expressed in the cytoplasm of both substate 1 and substate 2 cells, but not in the nucleus (Fig. 3b). TWIST was detected in the cytoplasm and nucleus of almost all cells and showed lower expression in substate 2 cells than in substate 1 cells (Fig. 3b). Importantly, ZEB1 was expressed only in the nucleus of substate 2 cells (Fig. 3b). The expression of the E-cadherin gene is negatively and directly regulated by numerous transcription factors, including ZEB1^29,30^. Thus, ZEB1 expression may repress E-cadherin and induce the endothelial-to-mesenchymal transition in substate 2 cells (Fig. 3).

### Substate 2 was an ectodermally biased pluripotent substate

**Figure 4:**
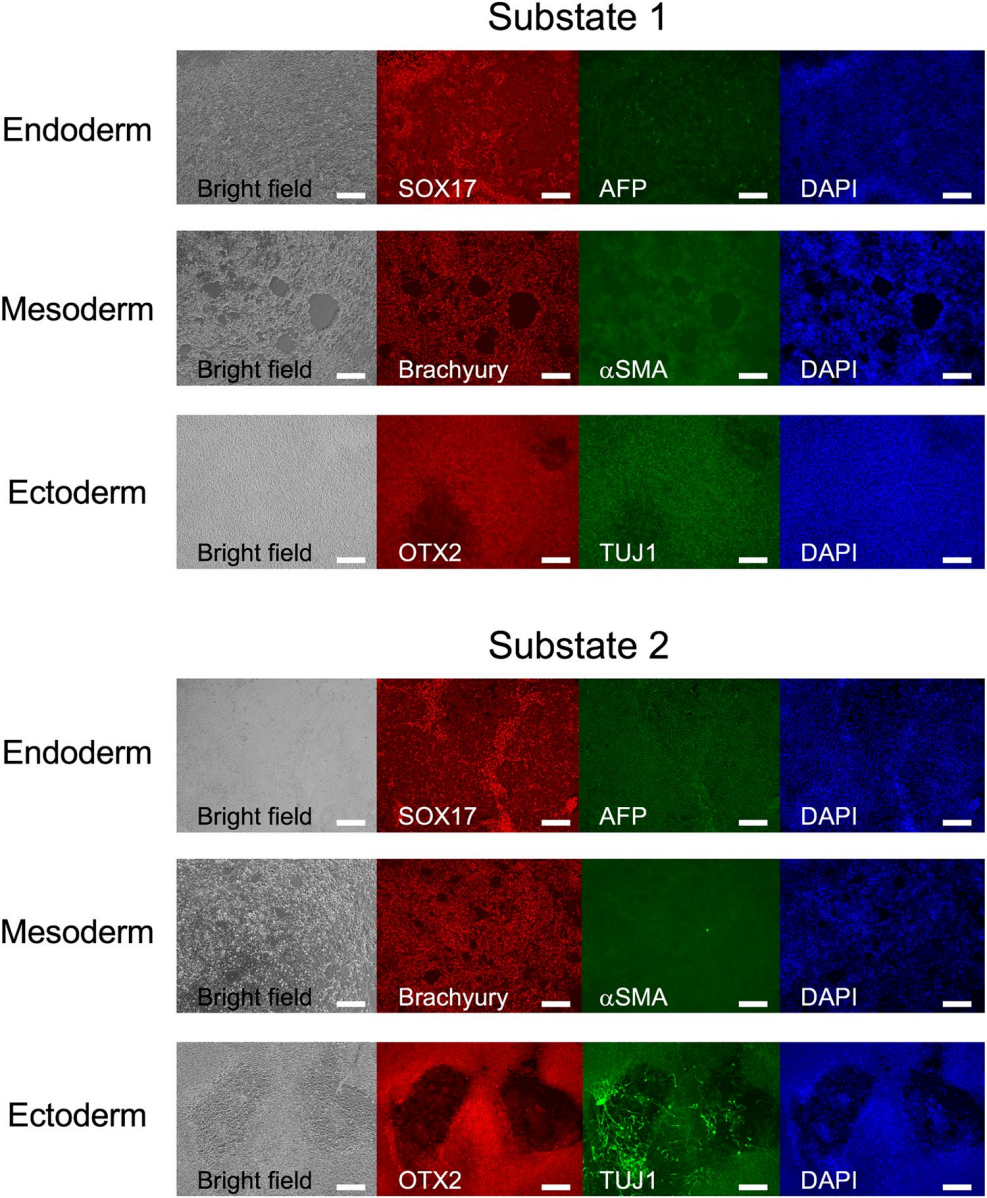
Immunohistochemical analysis of three germ layer markers in substate 1 and substate 2 of H9 cells. Immunohistochemistry with permeabilisation using antibodies for endoderm (SOX17 and AFP), mesoderm (brachyury and αSMA), and ectoderm (OTX2 and TUJ1) markers. Nuclei were counterstained with DAPI. The scale bar represents 200 µm.

To investigate differences in trilineage differentiation potential, substate 1 and substate 2 cells were concentrated by manually removing other cells and induced to differentiate into the three germ layers using a STEMdiff Trilineage Differentiation Kit. The induced cells were immunostained using antibodies for endoderm markers (SRY-box 17 [SOX17] and alpha-fetoprotein [AFP]), mesoderm markers (brachyury and α-smooth muscle actin [αSMA]), and ectoderm markers (orthodenticle homeobox 2 [OTX2] and neuron-specific class III beta-tubulin [TUJ1]) (Fig. 4)^31,32^. Endoderm- or mesoderm-induced cells of both substate 1 and substate 2 expressed SOX17 or brachyury in the nucleus and AFP or αSMA in the cytoplasm, respectively (Fig. 4). There were only minor differences in the expression levels of these targets in substate 1 and substate 2 cells. OTX2 expression in the nucleus did not differ between ectoderm-induced substate 1 and substate 2 cells (Fig. 4). Interestingly, TUJ1 was detected in the cytoplasm in ectoderm-induced cells of both substate 1 and substate 2, although only substate 2 cells formed neuron-like structures (Fig. 4). These results indicated that both substate 1 and substate 2 cells were able to differentiate into the three germ layers, i.e., endoderm, mesoderm, and ectoderm. Because ZEB1 controls neuron differentiation through transcriptional activation^33^, substate 2 cells expressing ZEB1 may differentiate more easily into ectoderm than substate 1 cells. In other words, substate 2 cells were ectoderm-biased pluripotent cells. Li *et al*. suggested that Matrigel induces ectoderm differentiation of embryoid bodies^23^. Wrighton *et al*. also reported that Matrigel inhibits mesendoderm differentiation of pluripotent stem cells^34^. These findings and our study indicated that Matrigel is an ectoderm-biased matrix.

### Ectoderm-biased substate 2 cells exhibited neural differentiation signals

**Figure 5:**
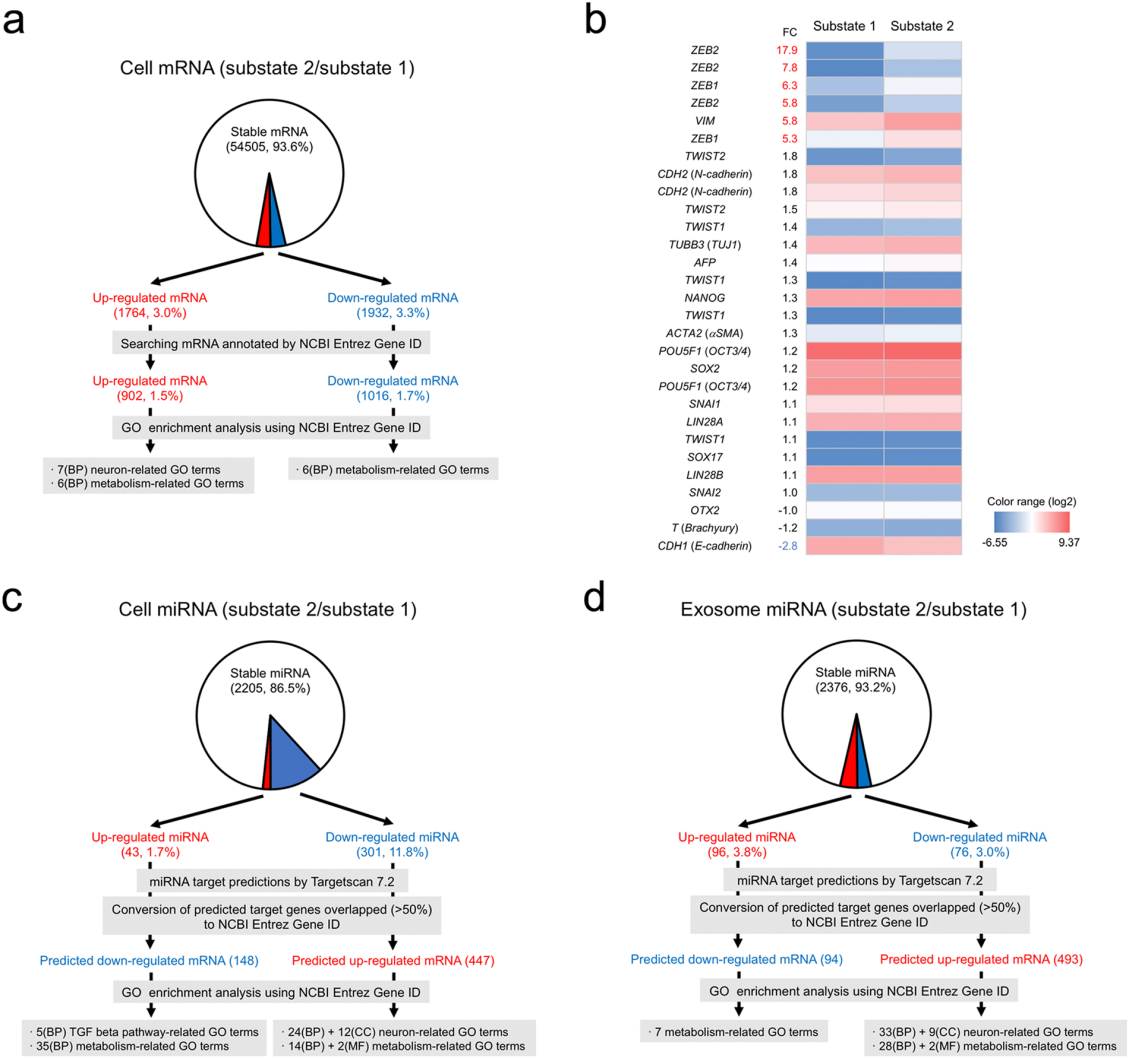
mRNA and miRNA expression analysis for cells in substate 1 and substate 2. (**a**) mRNA microarray analysis. Analysis of significance was performed using Z-score of substate 2 versus substate 1 (see Supplementary Fig. S1). The symbols of the obtained mRNAs were converted to NCBI Entrez Gene IDs by BioMart based on GRCh38.p12. GO enrichment analysis was performed using the IDs (false-discovery rate [FDR] ≤ 0.05, fold enrichment ≥ 1.5; see Supplementary Tables S2–S3). (**b**) Heat map showing pluripotency, epithelial-to-mesenchymal transition, and trilineage markers. The map was constructed using the mRNA microarray data of pluripotency markers (*CDH1* [E-cadherin], *CDH2* [N-cadherin], *LIN28A*, *LIN28B*, *NANOG*, *POU5F1* [*OCT3/4*], and *SOX2*), epithelial-to-mesenchymal transition markers (*SNAI1*, *SNAI2*, *TWIST1*, *TWIST2*, *ZEB1*, *ZEB2*, and *VIM*), and trilineage markers (*ACTA2* [*aSMA*], *AFP*, *OTX2*, *SOX17*, *T* [brachyury], and *TUBB3* [*TUJ1*]) (see Supplementary Table S4). The red and blue coloured fold change (FC) numbers indicate significantly higher and lower expression in substate 2 than in substate 1, respectively. (**c** and **d**) miRNA microarray analysis of the cells (**c**) and exosomes (**d**). The miRNAs that showed a 2-fold difference in expression between substate 1 and substate 2 cells were subjected to analysis of significance using paired t-test with multiple testing correction (q < 0.05; Supplementary Tables S5–8). The miRNA target prediction was performed by Targetscan 7.2. The predicted up- and downregulated mRNAs were derived from the down- and upregulated miRNAs, respectively (see Supplementary Tables S9–S12). The symbols were converted to NCBI Entrez Gene IDs using BioMart based on GRCh38.p12 (see Supplementary Tables S9–S12). GO enrichment analysis was performed using the IDs of genes targeted by more than 50% miRNAs (false-discovery rate [FDR] ≤ 0.05, fold enrichment ≥1.5; see Supplementary Tables S13–S16).

We established ectoderm-biased substate 2 cells, which tended to differentiate into ectoderm. To explore new biomarkers for the ectoderm-biased pluripotent substate, a comparison of global gene expression profiles between substate 1 and substate 2 cells was performed by DNA microarray analysis using Z-score (Supplementary Table S1 and Supplementary Fig. S1). We found 1764 and 1932 mRNAs showing significant up- or downregulation in substate 2 cells compared with that in substate 1 cells, respectively (Figs 5a and S1). After conversion of HGNC symbols to NCBI Entrez Gene IDs, 902 upregulated and 1016 downregulated mRNAs were obtained (Fig. 5a). We then performed gene ontology (GO) enrichment analysis using the NCBI Entrez Gene IDs (false-discovery rate [FDR] ≤ 0.05, fold enrichment ≥ 1.5), and seven neuron-related GO terms were specifically detected in biological process in the upregulated mRNAs, as expected (Fig. 5a and Supplementary Tables S2–S3).

Next, we analysed pluripotency, epithelial-to-mesenchymal transition, and trilineage markers, as described in Fig. 5b and Supplementary Table S4. E-cadherin and vimentin were expressed in substate 1 and substate 2 colonies, respectively, as shown in Fig. 3. As expected, *CDH1* (E-cadherin) and *VIM* (vimentin) were expressed at significantly higher levels in substate 1 and substate 2 cells, respectively (Fig. 5b). There were no significant differences in expression between substate 1 and substate 2 cells in pluripotent genes (*POU5F1*, *NANOG*, *SOX2*, *LIN28A*, and *LIN28B*), endoderm markers (*SOX17* and *AFP*), mesoderm markers (*T* and *ACTA2*), ectoderm markers (*OTX2* and *TUBB3*), and epithelial-to-mesenchymal transition markers, except for *ZEB1* and *ZEB2* (*SNAI1*, *SNAI2*, *TWIST1*, *TWIST2*, and *CDH2*; Fig. 5b). Importantly, expression levels of *ZEB1* and *ZEB2* were significantly higher in substate 2 cells than in substate 1 cells (Fig. 5b). The high expression of *ZEB1* mRNA in substate 2 cells may promote ZEB1 protein expression in the nucleus (Fig. 3b). These results indicated that ectoderm-biased substate 2 cells were undifferentiated, but exhibited neural differentiation signals. Unfortunately, approximately 1000 biomarker candidates for ectoderm-biased pluripotent substate were detected.

### miRNA analysis showed that substate 2 cells exhibited neural differentiation signals, similar to mRNA analysis

Not only mRNA but also miRNAs show promise as biomarkers for various diseases and cell quality evaluation^12,16,18^. Moreover, miRNAs derived from serum- and cell culture medium-exosomes could facilitate the noninvasive assessment of many diseases and cell qualities^16–18^. To explore new miRNA biomarkers for the earliest stages of ectodermal differentiation, we compared comprehensive expression profiles between substate 1 and substate 2 using miRNAs for cells and exosomes (Supplementary Table S1). miRNAs that showed 2-fold differences in expression between substate 1 and substate 2 cells were analysed. Significance analysis showed that 43 and 301 cell-derived miRNAs were upregulated and downregulated, respectively, in substate 2 cells compared with those in substate 1 cells (Fig. 5c and Supplementary Tables S5–S6). Moreover, we also identified 96 and 76 exosome-derived miRNAs that were upregulated and downregulated, respectively, in substate 2 cells compared with those in substate 1 cells (Fig. 5d and Supplementary Tables S7–S8).

miRNA target prediction was carried out by performing a keyword search in TargetScanHuman 7.2 (http://www.targetscan.org/vert_72/) using miRNA symbols to predict up- and downregulated mRNAs from the down- and upregulated miRNAs, respectively. The numbers of predicted up- and downregulated mRNAs targeted by more than 50% of the miRNAs were 447 and 148 or 493 and 94 in cells or exosomes, respectively, from substate 2 (Fig. 5c,d and Supplementary Tables S9–S12). GO enrichment analysis was performed using NCBI Entrez Gene IDs (Supplementary Tables S13–S16). Overall, 24 and 12 neuron-related GO terms were detected in biological processes and cellular components, respectively, for the predicted upregulated mRNAs derived from cell miRNAs (Fig. 5c and Supplementary Table S14). GO enrichment analysis of the predicted upregulated mRNAs derived from exosome miRNAs also showed 33 and nine neuron-related GO terms in biological process and cellular component, respectively (Fig. 5d and Supplementary Table S16). Five transforming growth factor (TGF)-β pathway-related GO terms were detected in biological process of the predicted downregulated mRNAs derived from cell miRNAs (Fig. 5c and Supplementary Table S13). Notably, ectoderm is differentiated from human and mouse ES cells in the absence of a TGF-β signal^35^. Accordingly, these results indicated that substate 2, which tended to differentiate into neurons, was characterised by specific expression patterns of mRNAs and miRNAs. However, in each analysis of cellular and exosomal miRNA microarrays, it was difficult to limit the number of biomarker candidates for the earliest stages of ectodermal differentiation.

### Integrated analyses of mRNA and miRNA microarrays of substate 1 and substate 2 cells

**Figure 6:**
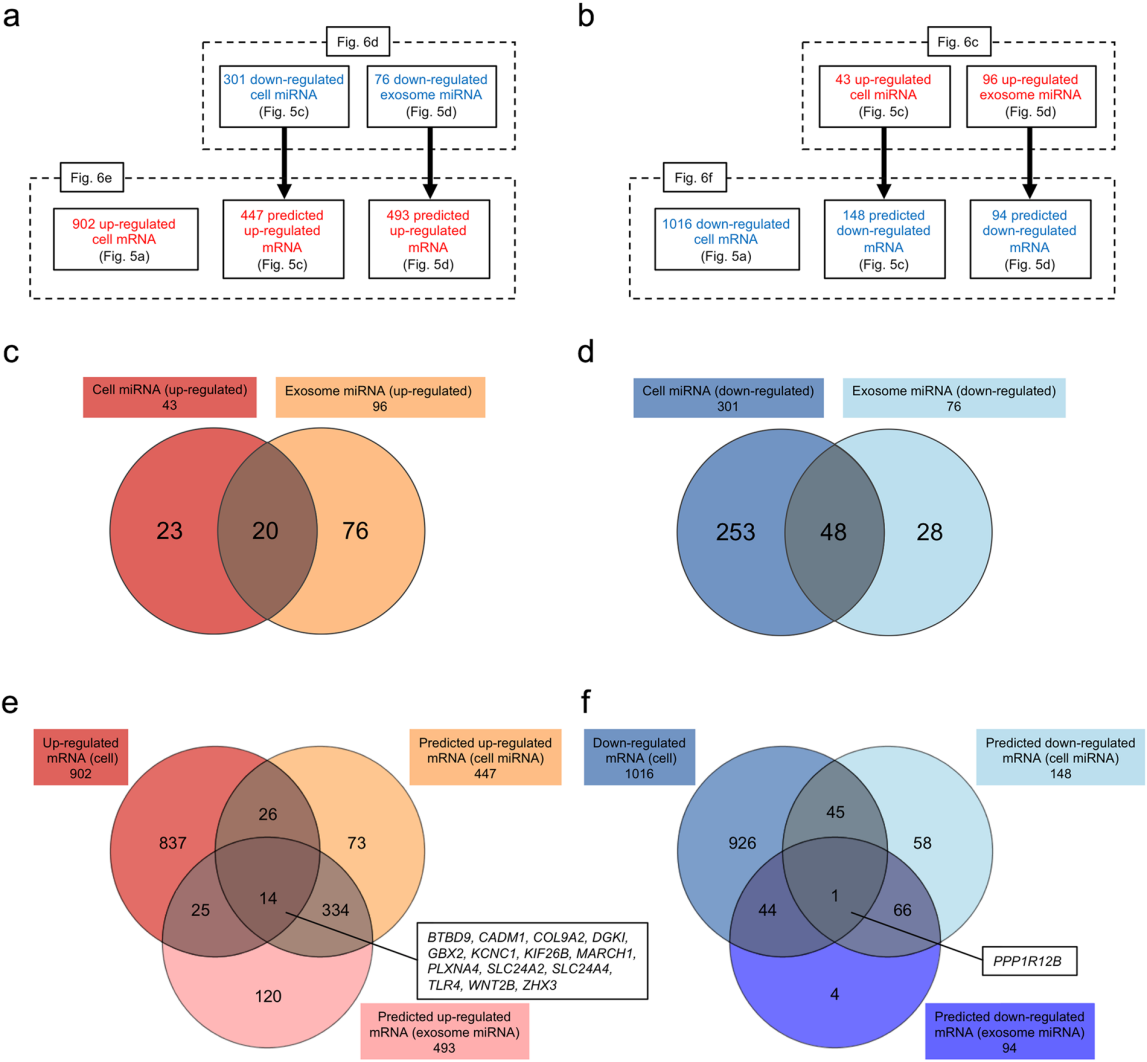
Integrated analysis mRNAs and predicted mRNAs targeted by miRNAs between substate 1 and substate 2. (**a**,**b**) Schematic diagram of integrated analysis of mRNA and miRNA microarrays. (**c**) Venn diagram of upregulated miRNAs from cells and exosomes. The miRNAs used are indicated in Fig. 5c,d and in Supplementary Tables S5 and S7. The upregulated miRNAs common to the cells and exosomes are shown in Supplementary Table S17. (**d**) Venn diagram of downregulated miRNAs from cells and exosomes. The miRNAs used are shown in Fig. 5c,d and in Supplementary Tables S6 and S8. The downregulated miRNAs common to the cells and exosomes are shown in Supplementary Table S18. (**e**) Venn diagram of upregulated mRNAs and predicted upregulated mRNAs targeted by downregulated miRNAs. The mRNAs in Supplementary Fig. S1, Supplementary Table S10, and Supplementary Table S12 were used. The upregulated mRNAs common to upregulated cell mRNAs and predicted upregulated mRNAs from miRNAs of cells and exosomes are shown in Supplementary Table S19. (**f**) Venn diagram of downregulated mRNAs and predicted downregulated mRNAs targeted by upregulated miRNAs. The mRNAs in Supplementary Fig. S1, Supplementary Table S9, and Supplementary Table S11 were used. The downregulated mRNAs common to downregulated cell mRNAs and predicted downregulated mRNAs from miRNAs of cells and exosomes are shown in Supplementary Table S20.

The ectoderm-biased substate 2 cells and substate 1 cells could differentiate into the three germ layers; therefore, these cells may have a few differential gene expression patterns. Integration of mRNA and miRNA approaches may provide new candidates for highly reliable biomarkers^19,20^. Accordingly, we attempted to conduct integrated analysis of mRNA and miRNA, as shown in Fig. 6a,b. We first discovered 20 upregulated and 48 downregulated miRNAs common to cells and exosomes in substate 2 using NCBI Entrez Gene IDs because miRNAs present in both cells and exosomes are reliable in microarray analysis (Fig. 6c,d and Supplementary Tables S17–S18). Twelve of the 20 upregulated miRNAs (*miR-105-5p*, *miR-125b-5p*, *miR-130b-5p*, *miR-135b-5p*, *miR-181a-5p*, *miR-181b-5p*, *miR-218-5p*, *miR-324-5p*, *miR-338-3p*, *miR-421*, *miR-592*, and *miR-1306-5p*) play important roles in neuron-related functions^36–47^. Stadler *et al*. previously reported that multiple members in a large cluster of miRNAs on chromosome 19, called the C19MC miRNA cluster, are enriched in undifferentiated human ES cells^48^. The C19MC miRNA cluster consists of 46 genes encoding 59 mature miRNAs^49^. Interestingly, 24 of 48 downregulated miRNAs in substate 2, which could maintain pluripotency, were members of the C19MC miRNA cluster. Next, 20 upregulated and 48 downregulated miRNAs were converted to predicted downregulated and upregulated mRNAs, respectively, using TargetScanHuman 7.2. We then searched for mRNA biomarker candidates based on a combination of mRNAs and predicted mRNAs targeted by miRNAs using NCBI Entrez Gene IDs. Fourteen upregulated mRNAs (*BTBD9*, *CADM1*, *COL9A2*, *DGKI*, *GBX2*, *KCNC1*, *KIF26B*, *MARCH1*, *PLXNA4*, *SLC24A2*, *SLC24A4*, *TLR4*, *WNT2B*, and *ZHX3*) and one downregulated mRNA (*PPP1R12B*) in substate 2 were obtained (Fig. 6e,f and Supplementary Tables S19–S20). Eleven of 14 upregulated mRNAs (*BTBD9*, *CADM1*, *DGKI*, *GBX2*, *KCNC1*, *KIF26B*, *PLXNA4*, *SLC24A2*, *SLC24A4*, *TLR4*, and *WNT2B*) were involved in neuron-related functions^50–60^. The single downregulated mRNA, *PPP1R12B*, has not been linked to any pluripotency-related function in ES and iPS cells to date.

### Biomarker candidates for the earliest stages of ectodermal differentiation

**Figure 7:**
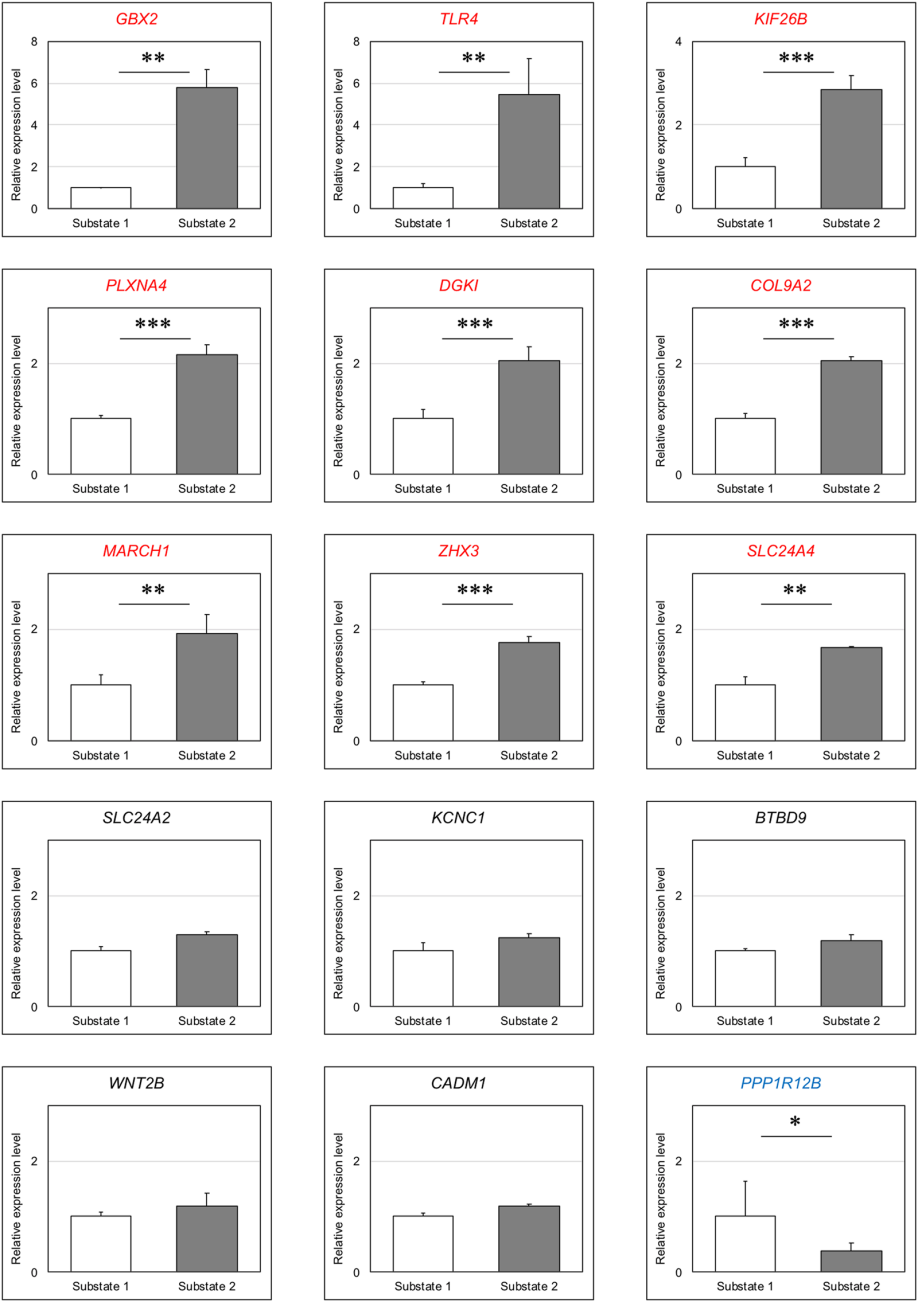
Expression analysis of biomarker candidates for the earliest stages of ectodermal differentiation. One-step real-time RT-PCR analysis of 14 upregulated mRNAs (*BTBD9*, *CADM1*, *COL9A2*, *DGKI*, *GBX2*, *KCNC1*, *KIF26B*, *MARCH1*, *PLXNA4*, *SLC24A2*, *SLC24A4*, *TLR4*, *WNT2B*, and *ZHX3*) and one downregulated mRNA (*PPP1R12B*) in substate 2 of integrated analyses of mRNA and miRNA was performed using RNAs derived from substate 1 and substate 2 cells. The expression of *GAPDH* was used for normalization. Values are the means ± SDs (n = 3). mRNAs that showed a 1.5-fold difference in expression between substate 1 and substate 2 cells were subjected to analysis of significance using unpaired two-tailed t-tests (*P < 0.1; **P < 0.05; ***P < 0.01). Red and blue gene symbols indicate upregulated and downregulated mRNAs, respectively.

To confirm the biomarker candidates obtained from integrated analysis of mRNA and miRNA, we performed one-step real-time RT-PCR analysis of 14 upregulated mRNAs (*BTBD9*, *CADM1*, *COL9A2*, *DGKI*, *GBX2*, *KCNC1*, *KIF26B*, *MARCH1*, *PLXNA4*, *SLC24A2*, *SLC24A4*, *TLR4*, *WNT2B*, and *ZHX3*) and one downregulated mRNA (*PPP1R12B*) using RNAs of substate 1 and substate 2 cells (Fig. 7). Nine of 14 upregulated mRNAs (*COL9A2*, *DGKI*, *GBX2*, *KIF26B*, *MARCH1*, *PLXNA4*, *SLC24A4*, *TLR4*, and *ZHX3*) were significantly upregulated in substate 2 cells compared with substate 1 cells. *PPP1R12B* expression tended to be higher in substate 1 cells than in substate 2 cells (*P* < 0.1). Allison *et al*. recently reported that endoderm-biased ES cells expressed higher levels of *GATA6*^10^, whereas few reports have described biomarkers that can be used to evaluate pluripotency during the earliest stages of differentiation. The nine upregulated genes were the most likely biomarker candidates for the earliest stages of ectodermal differentiation in pluripotent stem cells.

## Conclusions

Precise definition of the status of pluripotent stem cells during culture using biomarkers is essential for basic research and regenerative medicine. In this study, we first established the ectoderm-biased substate in H9 human ES cells. The ectoderm-biased substate was characterised by low and high expression of the pluripotency marker R-10G epitope and the mesenchymal marker vimentin, respectively. The ectoderm-biased cells could differentiate into all three germ layers. Finally, we identified nine upregulated mRNAs in the earliest stages of ectodermal differentiation by performing integrated analyses of mRNA and miRNA microarrays and qPCR analysis. Few reports have described biomarkers that can be used to evaluate pluripotency during the earliest stages of differentiation and in the germ layer-biased substates of human pluripotent stem cells. We provided new biomarker candidates that could be used in the earliest stages of ectodermal differentiation. Our findings may supply clues to develop high quality biomarkers to evaluate pluripotency.

## Materials and Methods

### Cell culture

H9 (WA09) human ES cells were obtained from the WiCell International Stem Cell Bank (Madison, WI, USA). H9 cells (passages 30–45) were maintained in TeSR-E8 (serum-free; cat. no. ST-05990; STEMCELL Technologies, Vancouver, BC, Canada) on Vitronectin XF (STEMCELL Technologies) under feeder-free conditions according to the manufacturer’s protocol provided by STEMCELL Technologies. H9 cells (passage 35) were cultured in TeSR-E8 on Matrigel (cat. no. 354230; BD Biosciences, NJ, USA) and then maintained until passage 65.

### Immunocytochemistry

Immunocytochemical analysis was performed as previously described^61^. The primary antibodies used were as follows: mouse anti-keratan sulfate (R-10G) antibodies (IgG; monoclonal; 1:150 dilution; cat. no. RIT-M001; Cosmo Bio, Tokyo Japan), rabbit anti-vimentin antibodies (IgG; monoclonal; 1:300 dilution; cat. no. ab92547; Abcam, Cambridge, UK), mouse anti-vimentin antibodies (IgG; monoclonal; 1:100 dilution; cat. no. SC-6260; Santa Cruz Biotechnology, Santa Cruz, CA, USA), mouse anti-SSEA-4 antibodies (IgG; monoclonal; 1:300 dilution; cat. no. MAB4304; Merck, Darmstadt, Germany), mouse anti-TRA-1-60 antibodies (IgM; monoclonal; 1:300 dilution; cat. no. MAB4360; Merck), mouse anti-E-cadherin antibodies (IgG; monoclonal; 1:100 dilution; cat. no. MA1-10192; Thermo Fisher Scientific, MA, USA), mouse anti-SSEA-1 antibodies (IgM; monoclonal; 1:250 dilution; cat. no. MAB4301; Merck), mouse anti-OCT3/4 antibodies (IgG; monoclonal; 1:300 dilution; cat. no. sc-5279; Santa Cruz Biotechnology), rabbit anti-NANOG antibodies (IgG; monoclonal; 1:800 dilution; cat. no. D73G4; Cell Signaling Technology, Danvers, MA, USA), rabbit anti-ZEB1 antibodies (IgG; monoclonal; 1:150 dilution; cat. no. ab203829; Abcam), rabbit anti-SNAIL1/2 antibodies (IgG; polyclonal; 1:200 diultion; cat. no. ab180714; Abcam), rabbit anti-TWIST antibodies (IgG; polyclonal; 1:300 dilution; cat. no. ab50581; Abcam), rabbit anti-SOX17 antibodies (IgG; polyclonal; 1:50 dilution; cat. no. PA5-72230; Thermo Fisher Scientific), mouse anti-AFP antibodies (IgG; monoclonal; 1:100 dilution; cat. no. MAB-1368; R&D Systems, Minneapolis, MN, USA), rabbit anti-brachyury antibodies (IgG; monoclonal; 1:500 dilution; cat. no. ab209665; Abcam), mouse anti-αSMA antibodies (IgG; monoclonal; 1:50 dilution; cat. no. M0851; DAKO, Glostrup, Denmark), rabbit anti-OTX2 antibodies (IgG; monoclonal; 1:200 dilution; cat. no. 701948; Thermo Fisher Scientific), and mouse anti-TUJ1 antibodies (IgG; monoclonal; 1:500; cat. no. MMS-435P; BioLegend, San Diego, CA, USA). The cells were incubated with primary antibodies diluted in 1% bovine serum albumin and 5% serum in phosphate-buffered saline (PBS) at 4 °C overnight. Secondary staining was performed with appropriate secondary antibodies, i.e., donkey anti-mouse IgG antibodies conjugated to Alexa Fluor 488 (IgG; polyclonal; 1:300 dilution; cat. no. A21202; Thermo Fisher Scientific), goat anti-mouse IgM antibodies conjugated to Alexa Fluor 488 (IgG; polyclonal; 1:300 dilution; cat. no. A21042; Thermo Fisher Scientific), and donkey anti-rabbit IgG antibodies conjugated to Alexa Fluor 594 (IgG; polyclonal; 1:300 dilution; cat. no. A21207; Thermo Fisher Scientific), for 1 h at room temperature. The samples were then counterstained with 4′,6-diamidino-2-phenylindole (DAPI; 1:1000 dilution; cat. no. D523; Dojindo, Kumamoto, Japan). The images were collected with a BIOREVO BZ-9000 fluorescence microscope (Keyence, Osaka, Japan). Based on bright-field and immunohistochemical observations, R-10G++ and vimentin− typical colonies and R-10G + and vimentin + colonies with wide intercellular spaces were designated as “substate 1” and “substate 2”, respectively.

### Flow cytometry

Flow cytometry was performed as previously described^62^. Briefly, H9 cells were digested with Accutase (cat. no. SF006; Millipore, Burlington, MA, USA) for generation of single-cell suspensions. The cells were incubated in MACS buffer (0.5% bovine serum albumin and 2 mM ethylenediaminetetraacetic acid in PBS) with mouse anti-vimentin antibodies (IgG; monoclonal; 1:33 dilution; cat. no. H00007431-M08; Abnova, Taipei, Taiwan) and then incubated with Alexa Fluor 488 donkey anti-mouse IgG antibodies (IgG; polyclonal; 1:300 dilution; cat. no. A21202; Thermo Fisher Scientific). Normal mouse IgG (1:33 dilution; cat. no. NI03; Merck) was used as an isotype control. Flow cytometry data were acquired using a SH800Z (Sony, Tokyo, Japan). The data were analysed using FlowJo v10 software (BD Biosciences).

### Cell reversibility

Substate 1 and substate 2 cells were separated from about 20% on the left and right sides of vimentin signals, respectively, using flow cytometry analysis of mouse anti-vimentin antibodies (IgG; monoclonal; 1:33 dilution; cat. no. H00007431-M08; Abnova). The separated substate 1 and substate 2 cells after the sorting were subcultured and then were analysed by flow cytometry analysis using mouse anti-vimentin antibodies (IgG; monoclonal; 1:33 dilution; cat. no. H00007431-M08; Abnova) and immunocytochemistry using mouse anti-R-10G antibodies (IgG; monoclonal; 1:150 dilution; cat. no. RIT-M001; Cosmo Bio) and rabbit anti-vimentin antibodies (IgG; monoclonal; 1:300 dilution; cat. no. ab92547; Abcam).

### Trilineage differentiation

H9 cells were differentiated to endoderm, mesoderm, and ectoderm using a STEMdiff Trilineage Differentiation Kit (cat. no. ST-05230; STEMCELL Technologies) according to the manufacturer’s protocol.

### DNA microarray and miRNA microarray

DNA microarrays were performed as previously described^62^. The mRNA was analysed using a SurePrint G3 Human GE Microarray Kit, 8 × 60 K Ver. 3 (cat. no. G4851C; Agilent, Santa Clara, CA, USA).

Cell culture medium was centrifuged to eliminate cell debris. Exosomes in the medium were precipitated using ExoQuick-TC (cat. no. EXOTC50A-1; System Biosciences, Palo Alto, CA, USA). miRNAs in cells and exosomes was extracted using an miRNeasy Mini Kit (cat. no. 217004; Qiagen, Valencia, CA, USA). The miRNA concentrations were calculated with an Agilent bioanalyzer using an RNA 6000 Pico Kit (cat. no. 5067-1513; Agilent). The samples were analysed using a SurePrint G3 miRNA Microarray Kit, 8 × 60 K (cat. no. G4872A; Agilent) and a miRNA Complete Labeling and Hyb Kit (cat. no. 5190-0456; Agilent). Arrays were scanned using a G2505C Microarray Scanner System (Agilent). The raw microarray data were submitted to the Gene Expression Omnibus (GEO) microarray data archive (http://www.ncbi.nlm.nih.gov/geo/) at NCBI (accession number: GSE123465, GSE123466, and GSE123470).

The data were analysed using Gene-Spring GX14.9.1 software (Agilent) after applying two normalisation procedures: (1) signal intensities of less than 1 were set to 1, and (2) each chip was normalised to the 75th and 99th percentiles of mRNA and miRNA measurements from that chip, respectively. Baseline transformation of these data was not performed. The signals for miRNAs derived from exosomes were corrected equivalent to 1 ng. Volcano plot, heat map, and clustering analyses were also performed using Gene-Spring GX14.9 software. GO enrichment analysis was carried out using PANTHER Overrepresentation Tests (http://geneontology.org/page/go-enrichment-analysis). miRNA target prediction was carried out by performing a keyword search in TargetScanHuman 7.2 (http://www.targetscan.org/vert_72/) using miRNA symbols.

### One-step real-time RT-PCR

Total RNAs were isolated from substate 1 and substate 2 cells using ISOGEN (cat. no. 319-90211; Nippon Gene, Tokyo, Japan). One-step real-time RT-PCR was conducted using total the RNAs with a One Step TB Green PrimeScript RT-PCR Kit (cat. no. RR066A; Takara, Kusatsu, Japan). The gene-specific primer pairs are shown in Supplementary Table S21. PCR amplification was performed on a StepOnePlus thermocycler (Applied Biosystems, Foster City, CA, USA) using the following thermal profile: 42 °C for 5 min, 95 °C for 10 s, and 40 cycles of 95 °C for 5 s and 60 °C for 31 s.

### Statistical analysis

Statistical analysis of microarray data except for Z-scores was performed using Gene-Spring GX14.9.1 software (Agilent). Z-score was analysed by calculating the standard deviations of log fold change results from microarray data using Excel software (Microsoft, Redmond, WA, USA). Unpaired two-tailed t-tests of qPCR data were performed using KaleidaGraph v4.5.2 software (Synergy Software, MN, USA).

### Ethics statement

This study was carried out in strict accordance with the National Institute of Advanced Industrial Science and Technology (AIST) guidelines for life science experiments (accreditation numbers 2016-099), and all human pluripotent stem cell experiments were approved by the Ministry of Education, Culture, Sports, Science, and Technology of Japan (MEXT).

## Supplementary information

Figure S1

Supplementary Tables S1-S21
