# Supplementary material for: mRNA and miRNA expression profiles in an ectoderm-biased substate of human pluripotent stem cells
Source: Sci Rep. 2019 Aug 15;9:11910. doi: 10.1038/s41598-019-48447-z (PMC6695399; doi:10.1038/s41598-019-48447-z)
Supplement: Supplementary file 1 — Figure S1 [file 41598_2019_48447_MOESM1_ESM.docx]

**mRNA and miRNA expression profiles in an ectoderm-biased substate of human pluripotent stem cells**

Shuuji Mawaribuchi^1^, Yasuhiko Aiki^1^, Nozomi Ikeda^1^, and Yuzuru Ito^1^

^1^Biotechnology Research Institute for Drug Discovery, National Institute of Advanced Industrial Science and Technology (AIST), Central 5, 1-1-1 Higashi, Tsukuba, Ibaraki 305-8565, Japan

**
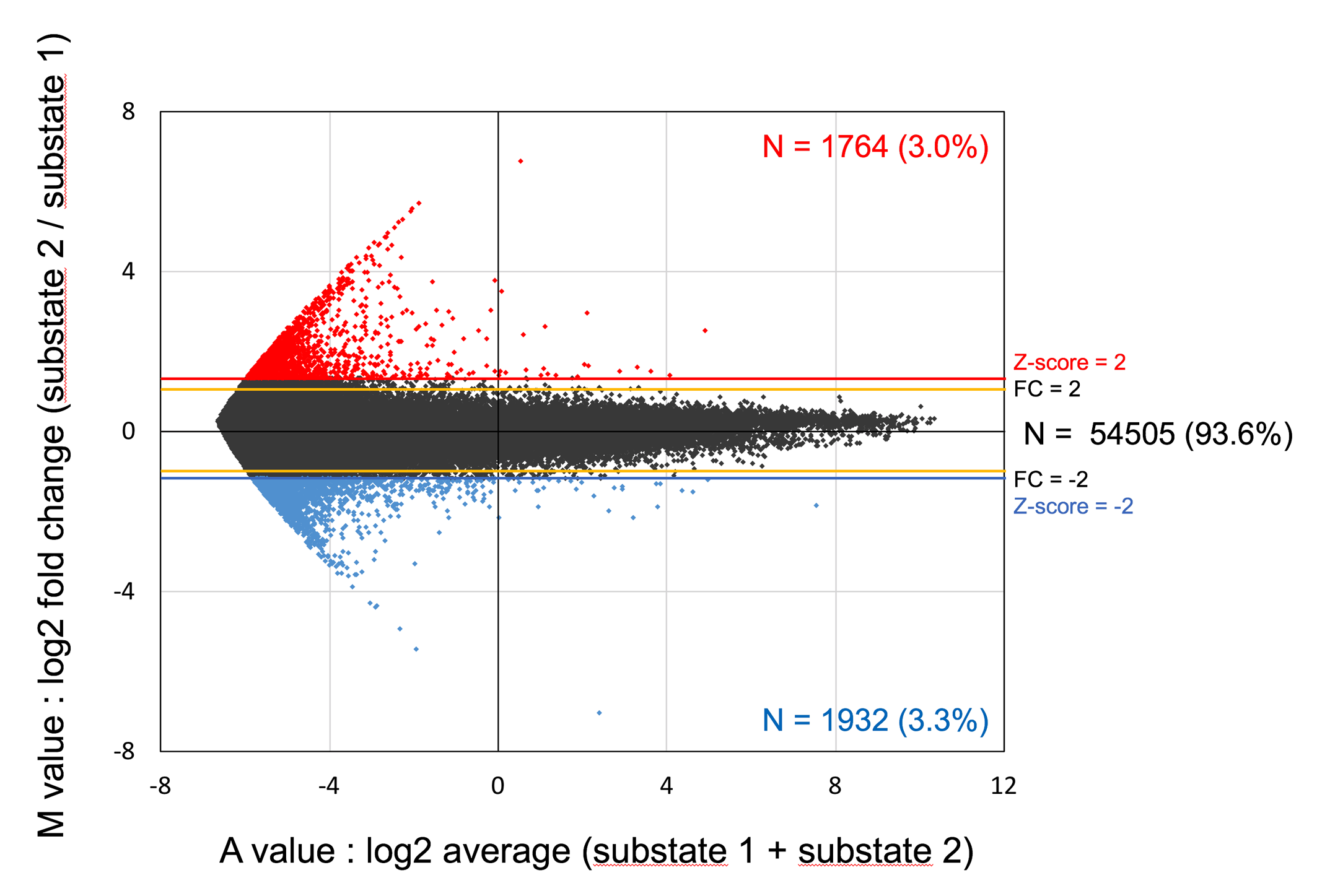
**

**Figure S1. MA plot of mRNA microarray data from substate 1 and substate 2.**

The Z-score of substate 2 versus substate 1 was calculated for statistical significance based on the mean and standard deviation (Z-score ≥ 2, fold-change ≥ 2.52; Z-score ≤ -2, fold-change ≤ -2.29). Red and blue plots indicated significantly higher and lower expression in substate 2 than in substate 1. N, number; FC, fold-change.
